# Complications of Open Reduction and Internal Fixation of Mandibular Condyle Fractures in Oman

**DOI:** 10.18295/squmj.12.2023.095

**Published:** 2024-08-29

**Authors:** Khamis M. Al Hasani, Abdulaziz A. Bakathir, Ahmed K. Al-Hashmi, Abdullah M. Albakri

**Affiliations:** 1 Dental and Maxillofacial Surgery Department, Sultan Qaboos University Hospital, Sultan Qaboos University, Muscat, Oman; 2 Dental and Maxillofacial Surgery Department, Al-Nahdha Hospital, Muscat, Oman; 3 Dental Department, Royal Oman Police Hospital, Muscat, Oman.

**Keywords:** Mandibular Fracture, Mandibular Condyle, Open Fracture Reduction, Complications, Facial Nerve Injuries, Oman

## Abstract

**Objectives:**

This study aimed to report the complication rate associated with open reduction and internal fixation (ORIF) of mandibular condyle fractures in Oman.

**Methods:**

This retrospective cohort study was conducted among patients who underwent ORIF of mandibular condyle fractures at Al-Nahdha Hospital and the Sultan Qaboos University Hospital in Muscat, Oman, from January 2008 to December 2020. Data collected included patient demographics, fracture aetiology, fracture side and type, surgical approach and recorded complications and outcomes.

**Results:**

A total of 68 patients (59 males and 9 females; mean age of 30.1 years) with 83 mandibular condyle fractures underwent ORIF during the study period. Subcondylar fractures were the most common type, occurring in 62.7% of patients, while bilateral fractures were observed in 21 (30.8%) patients. The most common surgical approach was retromandibular, used in 42.2% of patients. The overall complication rate was 42.6%, with the most frequently reported complications being transient facial nerve palsy (18.1%), malocclusion (14.7%) and restricted mouth opening (10.3%). Subsequent surgical interventions to correct malocclusion were performed in 6 cases. There was no statistically significant association between the overall complication rate and the patients’ clinical characteristics.

**Conclusion:**

Although ORIF of mandibular condyle fractures generally offers favourable outcomes, it carries a risk of complications.

**Advances in Knowledge**

*- Our findings indicate an overall complication rate of 42.6%, with transient facial nerve injury (18.1%) and malocclusion (14.7%) being the most common complications observed among the 68 patients treated*

**Application to Patient Care**

*- The findings of this study will serve as a reference during the process of obtaining informed consent from patients about to undergo open reduction and internal fixation (ORIF)*.
*- This study represents a continued movement towards the use of evidence-based medicine to discuss and explain outcomes, complications and risk-benefit ratios to patients before any procedure*.
*- The study’s findings will help surgeons assess risk, take preventive measures against complications and improve outcomes of patients undergoing ORIF of mandibular condyle fractures*.

Mandibular fractures are the second most common type of facial fracture, following nasal bone fractures, with mandibular condyle fractures accounting for 17.5–52% of all mandibular fractures.^1^–^4^ Management of condylar fractures (CFs) may involve conservative treatment, closed reduction or open reduction and internal fixation (ORIF).^2^^,^^3^^,^^5^^,^^6^ There is generally no clear consensus on the appropriate clinical indications for ORIF of CFs, except in cases involving reduction in ramus height, bilateral CF, severe displacement and dislocation.^5^–^8^

Closed reduction of CF is considered safe, with a predictably good outcome and minimal complications. Conversely, ORIF offers rapid restoration of function but is more technically demanding and associated with a higher risk of surgical complications.^1^^,^^3^–^7^ These complications may be related to factors such as the type of fracture, degree of segment dislocation, surgical approach and the surgeon’s skills and training.^1^^,^^2^^,^^5^ Over the past two decades, ORIF of CFs has gained popularity due to advancements in osteosynthesis materials, improved surgical skills and training and supportive evidence from scientific literature.^1^^,^^7^^,^^8^

Published literature on the complications of ORIF of CFs has identified various issues, including facial nerve injury, malocclusion, restricted mouth opening, osteosynthesis failure, infection, scarring, salivary fistula, bony complications and haemorrhage.^5^–^7^^,^^9^

In Oman, ORIF of CFs is becoming increasingly popular among oral and maxillofacial surgeons. To the best of the authors’ knowledge, there are no published studies on the complication rate of ORIF of CFs in Oman or the surrounding region. This study was conducted to report the complication rate of ORIF of mandibular CF in Oman and to identify the surgical approaches used and the outcomes for patients.

## Methods

This retrospective study was conducted at Al-Nahdha Hospital and Sultan Qaboos University Hospital (SQUH) in Muscat, Oman. It included all adult patients who presented with mandibular CFs and underwent ORIF from January 2008 to December 2020. Patients treated conservatively or through closed reduction, as well as those under the age of 16, were excluded from the study. Patient records were accessed and data were retrieved from the two electronic healthcare systems in Oman: Alshifa 3 Plus (Ministry of Health, Oman) for Al-Nahdha Hospital and TrakCare® 2018 (Unified Healthcare System, InterSystems Corporation, Cambridge, Massachusetts, USA) for SQUH.

The study’s data and variables include gender, age, mechanism of injury, type of CF according to the anatomic location (condylar head, condylar neck or subcondyle), fracture side (unilateral or bilateral), presence of other concomitant mandibular fractures, surgical approach, reported complications, need for re-operation and follow-up period with patient outcomes. Surgical approaches used for ORIF were classified as preauricular, retromandibular, anterior parotid transmasseteric rhytidectomy (APTMR), submandibular and endaural. Complications were categorised into nerve injury, malocclusion, restricted mouth opening, infection, haemorrhage, bony complications, hardware failure, scarring, salivary fistula and Frey’s syndrome.

Data collected during the study were entered into Microsoft Excel, Version 16.0 (Microsoft Corp., Redmond, Washington, USA) and statistical analysis was conducted using the Statistical Package for the Social Sciences (SPSS), Version 26 (IBM Corp, Armonk, New York, USA). Descriptive statistics summarised patient characteristics. Continuous variables were presented as mean ± standard deviation, while categorical variables were presented as frequency and percentage. The independent samples t-test was used for mean comparison between two groups. The association between two categorical variables was analysed using the Chi-squared test (Fisher’s exact/Likelihood ratio). A *P* value <0.05 was considered statistically significant.

Ethical approval for the study was obtained from the Research and Ethics Committee at Al-Nahdha Hospital (MOH/ANH/RC/10/5) and the Medical Research Ethics Committee at Sultan Qaboos University (MREC #2287) prior to commencement.

## Results

A total of 253 patients were diagnosed with mandibular CFs across the two hospitals during the study period. Among these, 68 patients with 83 CFs underwent ORIF and were included in this study. ORIF accounted for 26.9% of the total management of CFs. The study sample comprised 59 males and 9 females, with a mean age of 30.1 years. The mean postoperative follow-up period was 6 months (range: 1 month–5.8 years) [Table 1].

Of the 68 operated patients, 47 (69.1%) had unilateral fractures, while 21 (30.9%) had bilateral fractures. Forty-six (55.4%) CFs occurred on the right side and 37 (44.6%) occurred on the left. In terms of fracture subtype, subcondylar fractures were the most common (62.7%), followed by condylar neck (27.7%) and condylar head fractures (9.6%) [Table 1]. Additionally, 17 (25%) patients had isolated mandibular CFs, whereas 51 (75%) had compound mandibular fractures, with the symphysis and parasymphysis being the most common concurrent fracture sites.

The retromandibular approach was the most frequently used surgical approach for ORIF (42.2%). For subcondylar fractures, the retromandibular approach was utilised in 30 (57.7%) cases, the APTMR approach in 17 (32.7%) cases, the preauricular approach in 4 (7.7%) cases and the submandibular approach in 1 (2.2%) case [Table 1]. For condylar head fractures, only the preauricular approach was used in the 8 cases (100%). Condylar neck fractures were predominantly treated with the preauricular approach (10 cases, 43.5%), with an endaural approach used in 1 (4.3%) case. A statistically significant association was found between fracture site and surgical approach (*P* <0.05).

A total of 29 patients experienced at least one reported complication, resulting in an overall complication rate of 42.6% among the 68 patients. The most common encountered complication was transient facial nerve injury (18.1%), followed by malocclusion (14.7%) and restricted mouth opening (10.3%) [Table 2]. No statistically significant association was found between surgical complications and patients’ clinical variables [Table 3].

No cases of permanent facial nerve damage were reported. However, transient facial nerve weakness was observed in 15 cases. This weakness was associated with the preauricular approach in 9 cases, the retromandibular approach in 3 cases and the APTMR approach in 3 cases. All cases of transient facial nerve injury resolved completely within 5 months.

Malocclusion was the second most commonly reported complication, occurring in 10 cases. Of these, 4 patients had bilateral CFs. Additionally, 5 cases of malocclusion were associated with hardware failure.

Condylar resorption was observed in 2 cases of subcondylar fractures treated with the retromandibular approach. No significant association was found between condylar resorption and patients’ clinical variables, including gender, fracture site, side, malocclusion and surgical approach.

Intra-operative bleeding was reported in 5 cases. In 3 cases, bleeding was controlled with local measures such as packing, cauterisation and ligation. In 2 cases, emergency angiography was required to identify the source of bleeding, which were a dissecting aneurysm and maxillary artery pseudoaneurysm. Both cases were successfully managed by endovascular arterial stenting and embolisation, without further complications.

Frey’s syndrome was encountered in 4 cases, with 3 associated with the retromandibular approach and 2 with the preauricular approach. Additionally, 2 cases of salivary fistula were reported, both in association with the retromandibular approach used for subcondylar fractures.

Infection was reported in 7 cases: 3 presented as infected hardware and 4 presented as infected wounds. All infections occurred in association with subcondylar fractures, except for 1 which was associated with a condylar head fracture. Keloid scarring occurred in 3 cases, managed with steroid injections; 1 case required additional plastic repair.

Despite the 29 reported cases of complications, only 6 (20.6%) required further surgical intervention. These re-operated cases were related to malocclusion, hardware failure, infection and condylar resorption [Table 4]. Among the re-operated cases, 4 involved fractures in the subcondylar area.

## Discussion

ORIF is a crucial method for managing mandibular CFs. Despite its associated surgical complications, ORIF has gained global popularity over the past two decades.^5^–^8^ However, there is a significant variability in the reported complication rates for ORIF of CFs worldwide.^2^^,^^5^^,^^10^^,^^11^ A meta-analysis by Chrcanovic *et al*. reported a complication rate ranging from 27% to 67%.^2^ To the best of the authors’ knowledge, the current study is the first to address the complications of ORIF for CFs in Oman and the region, reporting an ORIF rate of 26.9%, which aligns with the rates found in other published studies.^10^^,^^11^

Various studies have documented a range of complications with differing occurrence rates related to ORIF of CFs, including facial nerve injury (0.3–48%), malocclusion (8.2% for bilateral condylar fractures), restricted mouth opening (3.9–20%), osteosynthesis failure (1.79%), scarring (10%), salivary fistula (2.3%) and condylar resorption (2.3%). The current study’s complication rates are consistent with those reported internationally.^1^^,^^4^–^6^^,^^8^

Facial nerve injury remains the most frequently reported complication of ORIF for CFs, with an overall rate ranging from 0.3–48.1%.^4^^,^^12^ Temporary nerve injury is more prevalent than permanent injury, which occurs at a very low incidence.^3^^,^^13^ A meta-analysis by Al-Moraissi *et al*. found a low risk of permanent facial nerve injury: 0.3% for the preauricular approach, 1.4% for the retromandibular approaches and 2.2% for the submandibular approach.^1^ The current study did not encounter any permanent facial nerve damage, supporting the notion that permanent facial nerve injury is not a major concern after ORIF of CFs.^1^^,^^14^

Al-Moraissi *et al*. also reported a 8–14% rate of temporary facial nerve injury associated with different surgical approaches.^1^ The submandibular approach, though less favourable due to limited accessibility to the condylar region, has been linked to an increased risk of temporary facial nerve damage, with reported rates ranging from 5.8% to 48.1%.^1^^,^^15^ This injury is often due to pressure on nerve branches during surgical retraction rather than direct nerve transection.^16^ The current study observed a temporary facial nerve weakness rate of 18.1%, with complete recovery within 5 months post-operatively. Notably, a higher incidence of temporary nerve injury was reported in the current study with the preauricular approach (40.9%), exceeding the 10% rate reported in previous meta-analyses.^1^^,^^13^^,^^15^ The preauricular approach provides direct access to the temporomandibular joint, facilitating good surgical access for condylar head and neck fractures.^1^^,^^15^ Al-Moraissi *et al*. highlighted excessive traction during this approach as a risk factor for facial nerve injury.^3^ Published data on facial nerve injury related to the retromandibular approach reported a slightly higher incidence of temporary facial nerve injury (14.4–17.2%) and permanent injury (1.2%). Manisali *et al*. documented a 30% risk of facial nerve injury.^17^ However, the current study found a significantly lower rate of nerve complications (8.6%) with the retromandibular approach. The APTMR approach offers direct access to and visualisation of the condyle, with a lower risk of facial nerve damage, as noted by Narayanan *et al*.^15^ However, it may lead to complications involving the parotid gland, such as sialocele, salivary fistula and Frey’s syndrome.^15^ In the present study, the APTMR approach was associated with a 12.5% rate of temporary nerve complications.

Complications related to the parotid gland are known to occur during the surgical repair of mandibular CFs. A systematic review has reported an incidence of 2.3% for sialocele and 4.3% for salivary fistula.^15^ For example, Downie *et al*. described a case of sialocele and salivary fistula associated with the retromandibular approach, while Narayanan *et al*. reported 4 cases of salivary fistulas.^14^^,^^15^ In the present study, 3 cases of Frey’s syndrome and 3 cases of salivary fistula were observed, but no cases of sialocele were encountered. The salivary fistulae resolved spontaneously within a few weeks and Frey’s syndrome, which results from the aberrant regeneration of parasympathetic nerves, was successfully managed with intracutaneous botulinum toxin injections.

In the current study, 1 case of condylar resorption was noted. This phenomenon, where the condylar position changes and ultimately leads to resorption, is sometimes seen in cases with rigid fixation and increased functional loading.^18^^,^^19^

ORIF allows for early mobilisation, which is beneficial in preventing ankylosis.^19^ It has been suggested that prolonged immobilisation beyond 10 days can increase the risk of ankylosis following condylar head fracture repair.^20^ The current study encountered 2 cases of ankylosis as a complication of ORIF. Xiang *et al*. reported 26 cases of post-operative ankylosis among 492 CFs fixed with ORIF, primarily associated with condylar head fractures.^21^ The present study’s finding align with existing literature that describes ankylosis as an uncommon complication of ORIF, particularly for condylar head fractures.^20^^,^^21^

Hardware failure was observed in 6 cases (7.2%), with 3 involving fractured bone plates and the remaining 3 involving loose screws accompanied by bone plate infection. Furthermore, among these cases, 3 had ORIF with a single mini-plate and 3 cases with 2 mini-plates. This finding is consistent with studies by Bergh *et al*, Parascandolo *et al*. and Al-Saadi *et al*.^6^^,^^22^^,^^23^ In contrast, Ellis *et al*. did not report any instances of hardware failure or surgical site infections, suggesting variability in outcomes depending on surgical approaches and techniques.^9^^,^^13^

Bleeding complications association with CFs often result from direct injury to the pseudoaneurysm of the internal maxillary artery.^23^^,^^24^ In the current study, intra-operative bleeding was encountered and managed with packing and ligation. Two cases of perioperative bleeding were linked to vascular aneurysms and required emergency angiography to identify the source. These cases were effectively managed with endovascular arterial stenting and embolization, avoiding further complications.^23^

The management of bilateral CFs, whether to treat one or both condyles by ORIF, lacks universal consensus and shows varied outcomes.^2^ Ellis *et al*. and others have noted that bilateral CFs often lead to malocclusion, restricted mouth opening and an increased risk of open bite.^9^^,^^11^^,^^13^^,^^25^–^27^ In the current study, 26% of patients with bilateral CF developed post-operative malocclusion, compared to 12.7% in those with unilateral CF. Multi-centre prospective randomised studies have highlighted the complexity of managing bilateral CFs due to different mechanisms compared to unilateral fractures. Nonetheless, ORIF of bilateral CFs tends to result in better outcomes, particularly in terms of occlusion and mouth opening range.^9^^,^^11^^,^^25^–^27^ Al-Moraissi *et al*. found that ORIF improves occlusion compared to closed reduction.^3^ In this cohort study, intermaxillary fixation using a guiding elastic was used post-operatively in 8.3% of cases, leading to improved occlusal outcomes, aligning with results reported by Kotrashetti *et al*. and Hyde *et al*.^10^^,^^16^

Despite the generally positive outcomes of ORIF, secondary surgical intervention may be necessary to address complications. In the current study, 6 cases required re-operation due to persistent deranged occlusion related to hardware failure, infection and condylar resorption. Although many studies addressed the complications requiring secondary surgery, details on these cases are limited.^9^^,^^12^^,^^28^^,^^29^ Kumaran and Soh emphasized the importance of timely diagnosis and intervention.^28^ Malocclusion can be managed with various approaches, including occlusal equilibration therapy, orthodontics or surgical options such as subcondylar osteotomy, gap arthroplasty, condylectomy, orthognathic surgery and total temporomandibular joint arthroplasty.^26^^,^^28^

This study’s findings, while significant, are subject to limitations inherent to retrospective studies, such as small sample size, incomplete or inadequate clinical record documentation and variable follow-up periods. Additionally, the diversity and complexity of CFs, along with potential confounding factors such as concomitant fractures, surgeon’s skills level and surgery duration, warrant further research to analyse these variables and their impact on the complications and outcomes of ORIF.

## Conclusion

Although ORIF of mandibular CFs offers a favourable outcome, it carries a risk of complications, with transient facial nerve injury and malocclusion being the most common complications encountered. This study highlights the importance of careful surgical planning and technique to minimize these risks and improve patient outcomes.

## Figures and Tables

**Table 1:** Demographic and clinical characteristics of the included patients (N = 68)

Clinical Characteristics	n (%)
**Gender**	
Male	59 (86.8)
Female	9 (13.2)
**Age in years (mean ± SD)**	30.1 ± 11.3
**Mechanism of injury**	
Road Traffic Accident	45 (54.2)
Fall	24 (28.9)
Assault	4 (4.8)
Animal kicks	4 (4.8)
Sports injury	2 (2.4)
Gunshot	1 (1.2)
Others	3 (3.6)
**Fractured site**	
Condylar head	8 (9.6)
Condylar neck	23 (27.7)
Subcondyle	52 (62.7)
**Fractured side**	
Unilateral	47 (69.1)
Bilateral	21 (30.9)
**Surgical Approach**	
Retromandibular	35 (42.2)
Anterior Parotid Transmasseteric Rhytidectomy	24 (28.9)
Preauricular	22 (26.5)
Submandibular	1 (1.2)
Endural	1 (1.2)

**Table 2:** The total frequency of complications associated with open reduction and internal fixation of mandibular condylar fractures (N = 83)

Complications	n (%)
**Malocclusion**	10 (14.7)
**Hardware failure**	6 (7.2)
**Restricted mouth opening**	7 (10.3)
**Nerve injury**	
Transient facial palsy	15 (18.1)
Preauricular paraesthesia	1 (1.2)
**Intraoperative bleeding**	5 (6.0)
**Infections**	
Infected bone plate	3 (3.6)
Infected wound	4 (4.8)
**Bony complications**	
Condylar resorption	2 (2.4)
Ankylosis	2 (2.4)
**Frey's syndrome**	4 (4.8)
**Salivary fistula**	2 (2.4)
**Keloid scar**	3 (3.6)

**Table 3:** Association between overall complications of open reduction and internal fixation of mandibular condylar fractures and patients’ clinical characteristics (N = 68)

Variable	Complications, n(%)		*P* value*
	Absent (n = 39)	Present (n = 29)	
**Gender**			0.481
Male	35 (89.7)	24 (82.8)	
Female	4 (10.3)	5 (17.2)	
**Age (mean ± SD)**	29.72 ± 12.53	30.59 ± 9.47	0.756
**Mechanism** †	(n = 45)	(n = 38)	0.596
Road traffic accident	22 (48.9)	23 (60.5)	
Fall	14 (31.1)	10 (26.3)	
Assault	2 (4.4)	2 (5.3)	
Animal kick	2 (4.4)	2 (5.3)	
Sport injury	2 (4.4)	0 (0.0)	
Gunshot	1 (2.2)	0 (0.0)	
Others	2 (4.4)	1 (2.6)	
**Fractured site** †			0.462
Subcondylar	30 (66.7)	22 (57.9.8)	
Condylar head	5 (11.1)	3 (7.9)	
Condylar neck	10 (22.2)	13 (34.2.5)	
**Fractured side** †			0.120
Unilateral	30 (76.9)	17 (58.6)	
Bilateral	9 (23.1)	12 (41.4)	
**Surgical approaches** †			0.367
Retromandibular	20 (44.4)	15 (39.5)	
APTMR	14 (31.1)	10 (26.3)	
Preauricular	9 (20.0)	13 (34.2)	
Submandibular	1 (2.2)	0	
Endaural	1 (2.2)	0	

SD = standard deviation; APTMR = Anterior Parotid Transmasseteric Rhytidectomy

* Independent samples t-test, Chi-squared test (Fisher's exact/Likelihood ratio);

† These variables were calculated from 83 total fractures, with complications (n = 38) and without complications (n = 45)

**Table 4:** Association between complications and re-operated cases

Variable	Re-operated, n (%)		*P* value*
	No	Yes	
Malocclusion	6 (60)	4 (40)	0.003
Hardware failure	3 (50)	3 (50)	0.004
Restricted mouth opening	5 (71.4)	2 (28.6)	0.112
Transient facial palsy	13 (86.7)	2 (13.3)	0.296
Preauricular paraesthesia	0	1 (100)	0.072
Intraoperative bleeding	5 (100)	0	1.000
Infected bone plate	0	3 (100)	0.0001
Infected wound	1 (25)	3 (75)	0.001
Condylar resorption	0	2 (100)	0.004
Ankylosis	2 (100)	0	1.000
Frey's syndrome	2 (50)	2 (50)	0.025
Salivary fistula	1 (50)	1 (50)	0.140
Keloid scar	3 (100)	0	1.000

* Chi-squared test (Fisher's exact/Likelihood ratio), significance level at P <0.05.
